# The rise of injecting drug use in east Africa: a case study from Kenya

**DOI:** 10.1186/1477-7517-2-12

**Published:** 2005-08-25

**Authors:** Susan Beckerleg, Maggie Telfer, Gillian Lewando Hundt

**Affiliations:** 1 Public and Environmental Health Research Unit, London School of Hygiene & Tropical Medicine, London, UK; 2 Bristol Drugs Project, 11 Brunswick Square, Bristol, UK; 3 Social Sciences in Health, University of Warwick, UK

## Abstract

Studies on injecting drug use in East Africa are reviewed. The existingstudies document the spread of heroin injection in Kenya and Tanzania, both countries where HIV rates are high. No data from Uganda on injecting drug use was found by the authors. A case study of the growth of heroin injection in a Kenyan coastal town is presented. The need for needle-exchange programmes and other prevention services is discussed.

## Background

Although bearing the brunt of the AIDS epidemic, Africa has long been considered largely free of injection drug use. Notwithstanding the assessments of the UN International Drug Control Programme [1] 1, international organisations have been slow to recognise either the spread of heroin use in Kenya or the existence of injection drug use. The largely unheeded spread of injection drug use in East Africa has wide implications for public health in the region. Injection drug users (IDU) are a 'high risk' or 'core group' for HIV infection. Many IDU share needles and syringes as well as having unprotected sex, and have been identified as a 'bridging population', speeding the spread of HIV to the general population [2,3] and [4].

Heroin injection now appears to be occurring in most large towns of Kenya and Tanzania. A study of 336 heroin users in Nairobi, Kenya found that 44.9% were, or had been, injectors [5]. Of 101 current injectors, 52.5% were HIV positive. This compares with a 13.5% prevalence rate among heroin users who had never injected. Hepatitis C prevalence also varied dramatically, from 61.4% among current injectors to 3.8% for those who had never injected. A similar study for Mombasa, Kenya's second city and main port, has been planned, but at the time of writing (November 2003) is yet to be carried out. However the UNODC and WHO are carrying out research in 2003 to establish links between HIV and drugs, with a focus on injecting, at the Kenya Coast.

Recent assessments in neighbouring Tanzania have found heroin injecting to be spreading throughout the country. Hence, a rapid situation assessment carried out in five Tanzanian towns [6] found heroin to be a major concern in Arusha, Dar es Salaam and Zanzibar, to be emerging as problem in Mwanza, but not in Mbeya. Injection drug use was reported in all the study sites where heroin was in use. Similarly, a study of 624 young multi-drug (alcohol, cannabis, tobacco, heroin, Valium, khat) users in Dar es Salaam found that 75% of the sample were using heroin, and that 114 (18.3%) of the sample reported injecting drugs [7]. As many of the substances used by the 624 people interviewed are not usually injected, the percentage of heroin users injecting in Dar es Salaam will be considerably higher than is indicated by these data which are not disaggregated by substance.

Much less is known about injecting drug use in Uganda. Indeed, the UNODC covering Eastern and Southern Africa reports that there have been no drug assessments carried out in Uganda.

## Discussion

### Case Study: The Kenya Coast

The case study draws together information collected by SB and MT from 1995 to 2003 as part of their work with the Omari Project, a Kenyan organisation pioneering the adaptation of international best practice treatment models for heroin users to the local setting of Coastal Kenya [8] and [9]. In addition, SB collected data on heroin users and injecting patterns between 2000 and 2001, as part of a larger ESRC funded study of women heroin users and their reproductive health.

Heroin has been a street drug at the Kenya coast since the 1980s where its use has spread from a few large towns to many smaller settlements, including some rural villages. The increasingly easy availability of heroin is linked to the 1980s tourist boom when Italian investors set up businesses with local partners. The Swahili community were particularly affected because they were in the forefront of the tourist industry and came into direct contact with Europeans requesting heroin [10].

As part of the Omari Project activities, MT and SB made assessments of the drug situation at the Kenya coast between 1995 and 1998 [8]. Until 1999, inhalation of vapour or 'chasing' 'brown sugar' was the dominant mode of use and the majority of heroin users at the Kenya coast were not injecting. 'White crest', a substance said by users to come from Thailand, started being mentioned in 1998 in Mombasa. 'White crest' cannot be chased but is easily injected. In late 1999 'brown sugar' disappeared from smaller coastal towns of Malindi and Watamu, and was replaced entirely by 'white crest'. Many chasers of 'brown sugar' became injectors of 'white crest'. The move to injecting was precipitated by the changes in the heroin supply that occurred in 1999. The UNDCP has been aware of heroin trafficking though the region. The publication by the UNDCP of trafficking routes from South Asia coincided with the decline in importance of such supply channels and the introduction of 'white crest' [11].

The ESRC study focused exclusively on Malindi, a town with a population of about 100,000. Since the 1960s Malindi has been a tourist resort. However, the town is an old Swahili city-state, already well established when Vasco da Gama visited 500 years ago en route to India. The original inhabitants of the area are Swahili fisher-people and traders. They have been joined by migrants from the rural hinterland and from the Kenya highlands, as well as by a significant minority of Europeans who own property in the area and are Kenyan residents.

In Malindi the switch to injecting heroin occurred in an area with high HIV rates. Sentinel surveillance carried out in antenatal clinics in the district showed a rate of 10% of attendees being HIV positive [12]. This figure was an average obtained from rural and urban areas within the district. In 2001 local health officials estimated that the HIV prevalence rate in the town was approximately 20%. It appears that the rate has remained at this high level, as about 20% of VCT clients undergoing HIV testing in the three centres operation in the District in July 2003 were positive (personal communication, VCT worker).

## Esrc Study Methods

One objective of the ESRC study was to estimate the numbers of male and female users in the town in 2000 [13], while ethnographic fieldwork enabled a more in depth understanding of patterns of heroin use and the emerging sub-culture [14]. The methods used in the ESRC study to collect the findings reported here are summarised below:

### Estimating user numbers

Users known to SB through her work with the Omari Project were asked to provide estimates of numbers of male and female users and how many were injectors. The lists of male users were cross-checked in interviews with other users to assess their reliability and to provide a source of data on which to base estimates of the number of male users residing in the town. The lists containing names of women users were the starting point for the snowball sample.

Snowball sampling has been used in the UK to contact drug users not known to the treatment services. The technique involves using each individual in the sample as a sampling node to generate the next subject until the sample is exhausted [15] and [16]. Snowball sampling has a number of potential drawbacks. For example, secret drug users who buy their supplies from different sources and use heroin alone could be missed from a sample generated from individuals who are part of a separate user network. Networks of heroin users might be formed along ethnic or class grounds, with members of one network having little or no knowledge of users in other networks.

In Malindi, Swahili people were concentrated in the old town area, but also lived in most neighbourhoods of the town. In addition, non-Swahili Kenyans were part of the same network of Swahili users living in the old town, and visited the area to purchase and use heroin and to meet other users. Men and women from various ethnic groups typically use heroin together, thereby overriding Swahili and Kenyan norms of gender and ethnic-based social segregation. However, a second network of European, mostly Italian users, exists, but was considered beyond the scope of the study. No attempt was made to include them in the estimates. In Malindi, where there appears to be one network of local users, a snowball sample was a useful means of contacting women users with a view to estimating their total number in the town.

Starting with two women already known to SB, female users were asked to list other women users. These were contacted and in turn asked to provide the names of other women users. They were also requested to participate in the study and assurances of confidentiality were made. Women were contacted all over the town until no new names appeared. In many cases this involved two 'generations' of individuals, and sometimes three. Throughout the research, SB updated her information on the identity of known women users, but during about two years of fieldwork few new names appeared.

### (Participant)-Observation

Ethnographic research methods involving participant-observation that 'collect rich qualitative data' [17] enable researchers, who have gained the trust of groups of drug users, to observe their behaviour, hear about how they talk about drugs and join their social networks [18-21]. Observation is also a means of validating the accuracy of reported behaviour.

Between March 2000 and October 2001, participant observation and in-depth interviews amongst heroin users were carried out by SB, mostly during three main periods of fieldwork lasting between two to three months, but also during three shorter visits of between two to four weeks. Through initial contacts from amongst the users already known to SB or contacted with the assistance of a key informant, Ali, SB located 24 women users. These women were part of a bigger, predominantly male network of users. SB spent most of the time in the streets and alleyways where users congregate and also in the homes of about 20 female and male users, with whom she had built up rapport. She also conducted unstructured, in-depth one-to-one interviews with these users [13].

SB was able to observe heroin being smoked and injected and able to listen to discussion concerning raising the funds for and the purchasing of, heroin. She visited all areas of the town, but was most familiar with the old town area, where many of her initial key informants resided. Users acting as key informants were assured of confidentiality both for themselves and those that they named [22].

There was no option of working with drug agencies and organisations. However, SB already knew some heroin users through her connections with the Omari Project. At the time of the study reported here, the Omari Project had carried out some street work in the old town area and detoxified several local users at its small headquarters in a neighbouring town. Users were aware that SB was a member of the Omari Project and that a free residential service was opening shortly or had recently opened. Although SB explained that the study had no direct connection to the intervention activities of the Omari Project, users perceived her as somebody interested in their problems and who might be able to provide assistance. Simmons and Koester [23] report a similar situation in the US. Hence, SB was seen as a non-threatening, non-drug user and unlike Moore [18] in urban Australia, there was never any question that she should be a participant rather than a mere observer of heroin or any other drug use. As a woman in her forties SB was the same age as the parents of many of the users. Crucially, certain key users who enjoyed high status amongst their peers would vouch for her [24]. Those users who chose to talk to SB about their lives seemed to see her as a safe listener and keeper of secrets.

## Estimates of User Numbers and Injectors

Our estimate of the total number of heroin users Malindi was 600 in the year 2000. This estimate was made after considering the lists produced by the male users for the old town area, and also taking into consideration the reported similar concentrations of users in three other neighbourhoods of the town. We traced or were informed of 26 women users in the town. The number of hidden and therefore uncounted female users is difficult to access, but is probably small. Hence, there were an estimated 30 women heroin users living in the town in 2000. During the two years of fieldwork, the number of about 25 known women users remained constant: although some female users died, moved away, went to prison or stopped using, they were replaced by others starting heroin use or moving into the area. Internationally, women form a minority of those in touch with drug services, and there are estimated to be far fewer women than men using heroin [25].

The assessments also included estimates of the proportion of heroin users who injected. The first male key informant approached by SB estimated that 80% of users in the town inject. Of the 15 named women users on the list provided by one women user, nine women were injecting and six were using by 'cocktail' (heroin mixed with cannabis) or 'joint' (heroin mixed with tobacco). The user who provided the list, a 'cocktail' user who had never injected, expressed shock at the high proportion of injectors on her list. Like most users, she perceived injecting to be more harmful than other modes of administration.

Hence, the percentage of injectors is difficult to estimate. According to the key informants in 2000, over half of users are injectors. However, such informants were long-term users who were likely to know other long-term users who had moved from smoking to injecting. New people are constantly being recruited to the ranks of heroin users and in this setting the vast majority of them do not start out as injectors. The estimated proportion of injectors, based on the multiple sources of data, was 50% in the year 2000.

Since 2000, there is nothing to indicate that the number of heroin users living in the town is decreasing. The price of a sachet of heroin has remained stable at KSh100 (approximately US$1.3). In 2003, outreach work carried out by the Omari Project located groups of new users concentrated in a suburb of the town where recent migrants congregate, an area which is also home to a prominent family of drug dealers. Most of the new users were heroin smokers, yet to make the transition to injecting. However, other users known to the Omari Project have moved to injecting within the last two years.

## Heroin Injecting Culture

### Language

SB found that the terminology used by members of the sub-culture of heroin users changes rapidly, so outsiders cannot readily gain entry to this group. As Ramos [26] found amongst Chicanos, ability to converse in this semi-secret language confers an insider status. The use of obscure terminology also assists heroin users in conducting their illegal and socially sanctioned activities within the midst of mainstream society. Heroin users in this Kenyan setting use a mixture of Swahili and English loan words to talk about injecting in particular and heroin use in general [10]. Most of these terms are slang and are not readily understood by Swahili-speakers who are not part of the heroin-using sub-culture. However, some terms such as 'junkie', 'shoot' or 'shooter' are understandable to drug users throughout the English-speaking world. Other terms are common to networks of heroin users within East Africa. Indeed, many words, such as *tapeli *('scam') seem to originate from mainstream Tanzanian dialects of Swahili and have been diffused into Kenyan drug slang. Other terminology, such as *kubwenga *meaning 'to inject', or *noma *meaning 'a bad or dangerous incident' (such as being chased by the police), appears to be specific to the speech of heroin users at the Kenya Coast. However, such terms are likely to spread quickly into general street talk [27].

### Injecting practice

Heroin users in Malindi have developed techniques and social protocols of injecting. In late 1999, when many users in the town moved from chasing 'brown sugar' to injecting 'white crest', one man was said by informants to have taught injection techniques for a fee. When interviewed he confirmed his 'teacher' role, adding that he now regretted being party to a change in heroin use that increased harm to users.

As users switched to injecting, many paid the fee and learnt how to inject themselves. Others remained dependent on 'doctors', users who inject others for a fee. Being a 'doctor' confers status and can be a source of easy money. Users who cannot inject themselves report having to raise the money to purchase heroin as well as an additional KSh40–50, representing about a 50% mark up on the price of the drug. Users report that a 'doctor's' services are paid for in cash and not in the form of a share of the heroin to be injected.

### Injecting equipment

Needles and syringes are available to users in a few local pharmacies for between KSh5–10. Combined syringes and needles, designed for single use are not available and separate barrels and needles are purchased. Needles are large gauge 'blue' or 'green' needles. Their large size means that they are not ideal for injecting into small veins. Damage to veins, usually seen after several years of injection in the UK, was widespread among those who had been injecting for less than 6 months. This accelerates a move towards use of other injecting sites e.g. small veins in hands and feet, and sites in the groin where veins are in close proximity to an artery. These carry greater risks for the injector.

Most injectors use the same equipment to inject more than once, with some reporting using needles that have become rusty from being stored in damp hiding places. In addition, repeated use blunts the needle and eventually causes jamming of the syringe [28].

### Injecting technique and sites

'White crest' used for injection is usually mixed with cold tap water. One or more sachets of one tenth of a gram of heroin are placed in the syringe. The required amount of water is drawn into the syringe and the solution shaken and examined for colour and to see that the 'white crest' has dissolved. If the user is injecting into the arm, a piece of string or rubber, or a belt or headscarf is tied round the upper arm as a tourniquet. Once the user finds a vein the needle is pushed in, the pump of the syringe is drawn back so that it fills with blood. The tourniquet is untied. Heroin is not always injected into the arms. Other injection sites amongst this group that I have observed are the legs, feet and backs of hands. Users also report injecting into the neck, near eyebrows, the groin area and the penis.

Users 'flush' a number of times. This procedure involves drawing blood back into the syringe and 'flushing' it back into the bloodstream. When asked, users have differing views on 'flushing'. Some say that it is best to flush several times, but that excessive flushing is damaging to the veins and smacks of desperation. Users will talk disparagingly of their friends who flush too many times while claiming that they themselves 'only flush two or three times'. Others claim that it is fine to flush as often as 'feels right'.

### Getting, storing and disposing of needles and syringes

Needles and syringes are sold in Kenyan pharmacies for less than $0.10. However, some pharmacy salespeople refuse to sell injecting equipment to those they suspect are using illicit drugs. In Malindi, possession of used needles and syringes can lead to prosecution. Therefore, weighing up the relative risks of misplacing injecting equipment, another person borrowing it or using it because they mistake it for their own, as opposed to the danger of arrest for its possession, leads many injecting drug users (IDU) to decide not to carry injecting equipment on their person. Few IDU buy new equipment each time they use heroin, but conceal needles and syringes in locations where drugs are consumed. SB and MT have observed six or seven identical, unmarked syringes secreted under the eaves of a house near a major using location. SB has also watched one man wrapping his equipment in an old plastic bag and concealing it in weeds at the side of the path near his house. This policy may indeed be prudent. SB's key informant, Ali, explained how he was once chased by the police and subsequently arrested. The evidence against him was two sets of needles and syringes that the Police found concealed under a rock in the garden of his family home. The case went to court, but was dismissed when Ali argued that many people had access to the garden and there was no way of proving that they belonged to him. Of course, in other settings, blood tests or finger printing would have established ownership.

Used needles and syringes litter the floors of spaces frequented by injectors. One concerned old town resident collected a bag filled with discarded injecting equipment and left it on the doorstep of the main pharmacy supplying needles and syringes at that time. Nevertheless, many users display an almost complete lack of concern about the disposal of injecting equipment for which they have no further use. SB has seen them toss them into the grass beside a busy thoroughfare and throw them out of the windows of their homes.

### 'Partners'

Many users pair up with a 'partner' to raise cash, hang out and use together in a fashion similar to the strategies of Puerto Ricans living in the USA [28]. In Malindi, as elsewhere, for women who usually earn money through sex work, having a male partner can be a useful security measure. Mixed pairs are sometimes, but not always, sexual partners. Yet, the relationship is not primarily a sexual one, but is focussed on pairing up to support each other in the mutual aim of getting and using heroin, and same sex partners are numerous. Usually both partners are either smokers or injectors. If the pair are injectors, they may inject each other with the same or separate injecting equipment. Users all claim to, and appear to have, their own equipment.

### Munira and her injecting

Munira was a young woman of about nineteen years of age. MT and SB had known her for several years, since she was first starting out as a heroin smoker. She earned money as a sex worker and was frequently involved in violent quarrels with other users and her family. Often, she raised money and used alone, although she sometimes paired up with an older woman. A detailed description of her injecting practice is provided elsewhere [30]. Below, SB's edited field notes illustrate the ways that her injecting was becoming out of control.

#### 27.4.00

Ali says he saw Munira this morning waving a syringe around in the street and complaining that she has been sold whitewash. I saw Munira later and she confirmed the story about injecting whitewash. She explained that she did not bother to check the colour after adding water because she was in a hurry. When she got a vein, she pulled the syringe so got blood, but it would not push in. At home they gave her 100 Shillings to buy more heroin.

#### 11.5.00

Even other users are particularly concerned about Munira. When we were talking to her, she developed a breathing problem and complaining of pain in her ribs. She is now resorting to injecting in the palm of her hand.

#### 12.5.00

Munira followed Ali from near the premises of the main dealer, asking him to inject her in a ruined house nearby by the light of a candle. He told me the story and said that he refused.

#### 15.5.00

Munira was almost caught injecting in the morning. She saw the policeman coming and stuck the syringe inside her blouse making her bleed.

The following year Munira was arrested on a theft charge and a used syringe was found hidden in her hair. The theft charges were eventually dropped against her when it emerged that she was merely collecting her fee for sex work from her client who was still sleeping. Nothing more was heard about drug charges.

### Sharing

Although most Malindi users possess their own needles and syringes, sharing of injecting equipment occurs, as it is common amongst IDU in other settings [28,31,32]. Sharing can occur in a number of different ways, but does not appear to be perceived as a routine practice in Malindi.

Independently of each other, several users explained to SB the mechanism for sharing out a one-tenth measure of heroin. The powder is mixed with water and shaken in the syringe. The share, proportionate to the amount of money paid, is decanted into the plastic needle cover. It may then be drawn up into a second syringe. SB asked another informant, Ibrahim, how injectors share one sachet of heroin. He replied that with injecting as opposed to smoking, sharing one sachet is not done much. If it became necessary to share one sachet between two injectors, one sachet is put in the syringe, the water added, and then shares proportionate to the amount of money contributed are measured out into a second syringe belonging to the other person. Alternatively, the solution is put back into the original syringe once the other user has injected and rinsed out the equipment after use. The degree of safety or risk to HIV exposure will, of course, depend on a number of factors, including whether there are one or two sets of injecting equipment used, and if the equipment is new. These procedures are similar to the 'back loading' and 'frontloading' procedures described by injecting drug users in European and American settings [28,3].

Whilst these heroin users are aware that there may be health risks associated with sharing, they often seem to under-estimate the potential dangers and are vague about the illnesses that can be transmitted through injection sharing. For example SB asked Ibrahim why people did not like injecting together. He replied it was because of the illnesses they could catch from sharing equipment. When SB asked what these were, he mentioned 'AIDS, pneumonia, scabies, ringworm and problems inside the body'.

Although there has been no systematic campaign to inform them of the dangers of sharing equipment to inject illicit drugs, there have been a number of national AIDS prevention campaigns highlighting the possible dangers of acquiring HIV from used needles and syringes. Indeed, the ease by which injecting equipment can be purchased in private pharmacies is linked to HIV prevention initiatives. Over the years, the Omari Project, during counselling sessions, has also made efforts to point out the dangers of injecting.

### Precautions taken by Ali and Elaine

When SB first met Ali he was the long-term sexual and using partner of Elaine. Elaine came from a wealthy background in the Kenya highlands and was better educated than most heroin users in the town. She had formerly owned a business, but had fallen on hard times. For the last couple of years she had lived with Ali, who had until recently been a successful dealer. Elaine described herself as a 'junkie' but took various measures in an attempt at discretion. Hence, for a period of time in 2000 she always injected into her legs, so as to avoid having track-marks on her arms. The trade off was sores and wounds on her ankles. By July 2000, Elaine was injecting into her arms, but with great difficulty. When SB asked Elaine about injecting practices, she confirmed what Ali had previously told me. When she was using with Ali in his family home one of them would agree to mark the syringe by burning the end, so that they knew whose was whose. The problem was that Ali's two brothers were also using in the family home and they could not be sure if they had used their (Ali's and Elaine's) syringes. However, she and Ali tried to hide them in places where they would not be found, and anyway, usually bought new ones everyday – others were put aside for standby in case the pharmacy was closed. She said that sometimes people asked to use her syringe after her – she told them she had AIDS, but they would reply that it did not matter. She said that people generally had their own syringes, but did not know if they marked them to distinguish them from others.

### Social status and injecting

Injectors tend to be long-term users with high consumption levels. High consumption of heroin confers status, but only if used in a controlled fashion. Users should be able to raise or acquire money and drugs easily, have autonomy and avoid public displays of heroin use that indicate loss of self-control. Once the money to buy heroin is raised, it is preferable to have sufficient funds to buy one's own supplies without resorting to sharing with others, and also be able to inject oneself in a comfortable setting. High status users tend to inject themselves at home or at a friend's place. Some users are homeless and sleep on verandas, or in boats on the beach. Amongst homeless users, heroin use takes place in other locations, such as a derelict house or sometimes on the streets in the open. Injecting at home avoids inconvenience and reinforces the message that one has a home. Using alone denotes that one has sufficient funds, and is therefore status enhancing, while not having one's own injecting equipment and borrowing or stealing from another user denotes a lack of control over one's life and a lack of autonomy [13].

Many users who inject in semi-public settings are embarrassed by their injecting practices, perhaps sharing the widespread general aversion to needles and syringes or perhaps, because according to their own local knowledge, injecting denotes a deeper level of dependency than smoking. This embarrassment or shame concerning injecting practices is far from unique [28]. On the other hand, some users, like Munira and Ibrahim, seek out opportunities to inject in public settings or walk around the neighbourhood with a syringe sticking out of their arms. This public display is not status enhancing because it denotes a lack of self-control. Sitting or standing in the semi-conscious state (*kuyoyoma*) in a public place, is a practice that is looked down upon by many users.

By 2003 only one pharmacy in Malindi would sell needles and syringes to heroin users. There have been a number of deaths of Malindi users, all injectors or ex-injectors. Nevertheless, it can be expected that the majority were sufferers of AIDS. Although in Malindi, the subject of HIV and AIDS remains 'taboo' amongst users and non-users alike; those who shared injecting equipment with now deceased members of their network have expressed their fear and despair to members of the Omari Project that they are also HIV positive.

## Discussion

The permutations of sharing injecting equipment with single or multiple partners within different venues, such as the home, public spaces or a shooting gallery have implications for the spread of HIV [3,31]. In Europe and North America much work on HIV awareness has been carried out, and most IDU are aware that sharing injecting equipment is a very risky activity. However, sharing continues to occur, particularly between sexual partners and when a user is suffering from heroin withdrawal. Nevertheless, the easy availability of new needles and syringes through needles exchanges has done much to reduce levels of sharing equipment. The situation in Kenya is different.

There is limited awareness of the dangers of HIV infection from sharing injection equipment, and limited access to new or clean equipment. The widespread injection of heroin is a recent development, yet injecting practices have acquired social significance. This new culture of injecting heroin and the developing protocols of sharing have implications for the transmission of HIV. While high status individuals sometimes help out other users who are in withdrawal by providing a few free puffs of heroin, they would not routinely allow their equipment to be used or to share injected heroin with others. This emphasis on individual use, as opposed to sharing heroin and injecting equipment, is an aspect of mainstream Kenyan life where individual effort and enjoyment of the fruits of personal endeavour are the dominant survival strategy [33].

Heroin use in Coastal Kenya should be recognised as part of the process of economic and cultural globalisation. Although large scale trafficking in heroin between South Asia and East Africa is a recent phenomenon dating from the late 1970s, there is a long history of contact between nations bordering the Indian Ocean. In Kenya, heroin use is spreading to smaller towns and even remote villages. Hence, 'drug abuse' is reported to be 'rampant' in Mbajumwali [34], a small village on Pate Island within Lamu District on the North Kenya coast. This newspaper report confirms that heroin is easily available in Mbajumwali and the nearby bigger village of Kizingtini.

In other settings, the easy availability of new needles and syringes through needle exchanges has done much to reduce levels of sharing equipment, but this opportunity has not been available to injectors in Kenya. Studies from other parts of the world demonstrate that needle exchanges can reduce the transmission of HIV and other blood borne diseases [35-37], but that needle exchanges are most effective when part of 'an integrated set of diverse preventive measures' [38] as has been applied in Australia. Indeed, some studies in American and European cities demonstrate that needle and syringe exchanges have not prevented rises in HIV and Hepatitis B and C infections; such rises occurring among drug injectors in Vancouver [39], Montreal [40] and Amsterdam [41]. The reasons for the failure in prevention of HIV and Hepatitis B and C were the dilution of the impact of needle exchange schemes, due to an inadequate volume of syringes supplied (especially where cocaine injection was widespread as in Vancouver), insufficient focus on the risks of sharing injection paraphernalia, little impact on sexual behaviour and a non-interventionist ethos.

Developing needle exchange services in Kenya carries a clear responsibility to learn from such research. The findings of these studies indicate that the provision of adequate supplies of injecting equipment must be coupled with an interventionist ethos, whereby risk behaviour is reviewed regularly with injectors and strategies are developed to minimise these. This challenge is both exciting and immense.

## Conclusion

### Towards a Regional Response

Odek-Ogunde et al [5] found 44.9 % of heroin users in Nairobi had been or were injectors. In addition, 'white crest' is widely available in Nairobi and injection techniques are similar to those observed at the Coast (Odek-Ogunde, personal communication). Kenyan heroin users and development workers report that heroin is even more widely used and easily available in Nairobi and Tanzanian cities such as Zanzibar, Arusha and Dar es Salaam, than at the Kenyan Coast. The recent study from Dar es Salaam, Tanzania [7] indicates that injecting drug use is widespread in the Tanzanian capital. Similarly, the 2001 assessment of drug use in Tanzanian towns [6] found injecting drug use to be a matter of serious concern and advocated the setting up of needle and syringe exchange programmes. However, injecting drug use in Tanzania extends beyond the areas assessed in the 2001 situational analysis. For example, anecdotal reports by the Zanzibar Youth Forum (personal communication) indicate that rates of injecting are higher on the more remote island of Pemba than in Zanzibar town (the islands of Pemba and Unguja comprise Zanzibar, which is also the name of the main town on the island of Unguja).

Heroin users at the Kenya Coast, as elsewhere within the region, lack information about the dangers of injecting, the risks of sharing needles and syringes and unprotected sex. Odek-Ogunde has called for harm reduction measures and notes that 'the presence of IDUs in Kenya creates a three fold risk: the escalation of injection drug use, the potential of exacerbating the HIV/AIDS situation, and the creation of a source for other drug-related damage' [42].

Coastal Kenyan heroin injectors share injecting equipment and have sex with each other as well as with non-users. Their risky behaviour takes place in a setting where about 20% of the general population are estimated to be HIV positive [13]. The need for harm reduction initiatives amongst this high-risk group is clear. The findings of the study in Nairobi, which show that 1 in 2 injectors interviewed is HIV Positive, demand an urgent response [5]. Heroin users lack information about the dangers of injecting, of sharing needles and syringes, and of unprotected sex. Most East African towns have a lively sex industry and indeed the vast majority of women users work in this field.

Best practice from around the world demonstrates that HIV prevention through harm reduction measures should continue to target 'high risk groups' even when the epidemic has moved into the general population [2,4].

In November 2003, the Omari Project obtained funding from the UK Community Fund to open a drop-in centre in Malindi where harm reduction strategies, including needle exchanges will be pioneered. This offers an opportunity to curtail the transmission of HIV and other infections such as Hepatitis C, the prevalence of which is unknown in Kenya, although data from Nairobi indicates that both are very prevalent. The development of this intervention also offers the opportunity to learn from studies of needle exchange 'failures' in Europe and North America and to avoid repeating their unwitting mistakes.

The UK Department for International Development (DFID) has expressed interest in supporting such initiatives and extending them to other East African cities, and is providing funding to the Omari Project to assess injection drug use in Mombasa, with a view to developing appropriate harm reduction services, including a needle exchange. As in most parts of the world [29], functional needle exchange programmes will require careful advocacy work if the police and community are not to oppose their operation. The ESRC study in collaboration with the Omari Project, reported above, has already held a series of workshops with health workers and key community members in Malindi, where the dangers of injecting drug use and its relationship with HIV have been highlighted [14].
